# Determinants of breast screening participation using small-area data in South Australia: gaining past and future insights from geospatial evidence

**DOI:** 10.1007/s10552-025-02009-z

**Published:** 2025-05-14

**Authors:** Ming Li, Deborah van Gaans, Muktar Ahmed, Anh-Minh Nguyen, Michelle Reintals, Andrew Holmes, David Roder

**Affiliations:** 1 Cancer Epidemiology and Population Health, University of South Australia, Adelaide, South Australia Australia; 2 Public Health Information Development Unit, Torrens University, Adelaide, South Australia Australia; 3 BreastScreen SA, Adelaide, South Australia Australia

**Keywords:** BreastScreen SA, Health service, Geospatial analysis, Screening participation rate, Social determinants, Socio-economic disadvantage, Small area characteristics

## Abstract

**Purpose:**

To profile breast screening participation at small-area (SA2) level in South Australia (SA) and capture local variations in socio-economic factors, access to healthcare, and cultural influences screening behaviors in ways that larger administrative units might overlook.

**Methods:**

SA2 demographic (2016 Census) and breast screening data in SA (2014–2015) were linked and analyzed. The dependent variable, biennial screening participation (ages 50–74 years), was classified as “low” if below the SA-wide biennial participation rate of 58%. Independent variables included SA2-level sociodemographic factors (e.g., socio-economic status, residential remoteness, country of birth) derived from Census data. Stepwise multivariable logistic regression was used to estimate the adjusted odds ratios (aORs) for low screening participation associated with SA2 demographic characteristics.

**Results:**

BreastScreen SA participation for the 164 SA2 areas was 50.6%, ranging from 41.1% for ages 55–59 to 67.8% for ages 60–64. Indicators of low participation included disadvantaged socio-economic quintile (aOR increasing to 17.00, 95% CI 9.84–29.36 for quintiles 3–5 compared with the least disadvantaged quintile 1), non-metropolitan residence (aOR 4.94, 95% CI 2.30–10.60), and mortgage/rental stress in low-income households (aOR increasing to 6.59, 95% CI 3.34–13.00 for the third compared with first stress tertile). Areas providing more unpaid care support for disabled/aged people had reduced odds of low screening participation (aOR 0.41, 95% CI 0.24–0.70). Characteristics indicating low odds of low screening included a higher proportion of Australian born (tertile 2, aOR 0.52, 95% CI 0.30–0.88, and tertile 3, aOR 0.27, 95% CI 0.11–0.67).

**Conclusion:**

Further model that aims to improve breast screening participation need to be explored at both individual and SA2 levels. Potential cultural and linguistically diverse (CALD), Indigenous, and socio-economic indicators could be drawn from the newly available ABS-managed PLIDA platform. More contemporary SA2 and screening data should also be used for prospective evaluation.

**Supplementary Information:**

The online version contains supplementary material available at 10.1007/s10552-025-02009-z.

## Introduction

Breast cancer is the most common cancer recorded in Australian women by population-based cancer registries, accounting for an estimated 28% of new cancers in 2021 [1]. It is the second leading cause of cancer death in women, responsible for 14% of these deaths and is exceeded only by lung cancer [1]. Survival has increased by 43% in Australia since the mid-1990s, potentially due to early detection, improved breast cancer treatments, and contributions from ongoing research [1].

BreastScreen Australia launched a national population-based mammographic screening program circa 1991, with a principal target age range of 50–69 years and with biennial screening eligibility from 40 years of age. From 2013, the principal age target was extended to 50–74 years. The official national accreditation standard for population coverage by biennial breast screening has been 70% for ages 50–69 years since program inception [2], although no Australian jurisdiction has ever achieved that standard. The program is publicly funded and systematically invites eligible women using Medicare enrollment data. However, women may also undergo mammography outside the program through private providers or opportunistic screening by general practitioners, which is not systematically reported to BreastScreen [2].

Beneficial effects of breast screening have been demonstrated by the Australian Institute of Health and Welfare (AIHW), and other researchers in Australia [3]. Studies of screening through BreastScreen have been widespread among women screened at ages 50–69 years, with 60% having this history in New South Wales (NSW) and 53% in South Australia (SA) in the 24 months preceding their diagnosis [4, 5]. Breast screening among the ever screened was 30% less likely for cancers with regional spread at diagnosis, and 51% less likely for those with distant spread at diagnosis, than for localized cancers [4]. Early diagnosis associated with breast screening facilitated more treatment options, leading to a 2.5 times greater likelihood of breast-conserving surgery and other early treatments [6, 7].

The impact of breast screening on survival has been investigated in SA, indicating screening to be associated with the following: Lower breast cancer mortality [8], a higher cancer survival (and a dose–response relationship); and with women having their last breast screening within 2 years and 5 years of diagnosis having a 35% and 27% lower breast cancer mortality, respectively. These impacts were accompanied by an increasing use of conservative breast surgery [5]. Beneficial effects of breast screening on survival have also been indicated in Queensland, where women with breast cancer diagnosed through breast screening were found to have higher survival than those whose breast cancers were found at symptomatic presentation [9]. Also, a case–control study in Western Australia [10] and a national cohort study [11] pointed to beneficial effects of breast screening in lowering breast cancer mortality.

Breast screening participation rates have never achieved the national target of 70% at national or jurisdictional level [3]. This is despite the evidence of a beneficial effect of screening and increases in BreastScreen Australia program activity in women aged 50–74 years from 1.7 million screening episodes in 2014–2015 to 1.9 million in 2018–2019 prior to emergence of the COVID-19 pandemic [12]. A constraining factor was that service supply did not keep pace with population growth. Age-standardized participation rates remained steady at approximately 53% in 2014–2015 among eligible women aged 50–74 years, providing a baseline for understanding geographic disparities, though participation declined further to 50% nationally in preliminary 2021–2022 data post emergence of the COVID-19 pandemic [3]. In 2018–2019, the biennial participation rate for ages 50–74 years was 54% nationally and 58% in SA [4], with the SA figure ranked second to the Tasmanian participation of 60% [13]. SA’s comparatively lower rates may be partly attributable to its demographic and geographic characteristics, particularly the greater proportion of the population living in rural or remote areas, which may limit service accessibility. Additionally, national participation rates were generally lower for non-English-speaking women, and for those residing in remote and socio-economically disadvantaged areas [3]. Given these patterns, SA presents an important case for investigating how screening participation varies at a more granular spatial level.

To increase participation, the BreastScreen Australia endorsed National Accreditation Standards (NAS) for promoting high service quality and access, which contained added emphasis on screening promotion for Indigenous women, those from culturally and linguistically diverse backgrounds, and those having rural/remote residences and lower socio-economic backgrounds [3].

Exploring participation rates across small geographical areas was undertaken to identify areas where disparities existed and where customized strategies may be needed to increase equity, accessibility, and participation in breast screening. Identifying residential areas with low participation is considered important for framing initiatives to increase screening coverage and reducing inequities.

To date, studies focused on the effects of breast screening settings on participation have been limited in number, focusing on estimated travel distances from women’s residential postcodes to screening locations, with results presented by residential area and socio-demographic characteristics such as age, language spoken at home, education, employment, and motor vehicle ownership [14–16]. The Australia Bureau of Statistics (ABS) has developed statistical areas (SAs) to represent community groupings that interact together socially and economically based on population size, typically 3,000–25000 people, functional areas, growth, suburbs, other localities, and local government areas [17]. These boundaries are designed to reflect the socio-economic and geographic diversity within SA, capturing local variations that larger administrative regions might overlook. We have used these SA2 regions in this study as they are generally the smallest geographic areas for which area-based health and vital ABS statistics are made available, revealing localized disparities, although such statistics would also exist by smaller Mesh Blocks and SA1 areas [17].

A recent study of SA2 sociodemographic profiles indicated that ethnocultural characteristics were associated with geographical disparities in screening participation [18]. Similar SA-based breast screen participation and associated sociodemographic factors have not been investigated at an SA2 level [19]. We address this evidence gap by applying Andersen’s Healthcare Utilization Model [20], which conceptualizes healthcare use as a function of predisposing factors (e.g., age, education, cultural background), enabling resources (e.g., income, geographic accessibility), and perceived or evaluated need for care. This framework supports a structured exploration of how small-area sociodemographic contexts influence breast screening behavior and aligns with broader literature on equity in access to preventive health services. To generate actionable insights for prioritizing services and research, we use the Social Health Atlas as the data source [21] to identify sociodemographic indicators of low screening participation at the small-area level.

## Materials and methods

### Geocoded BreastScreen SA data

A total of 261,798 records, with names and other identifiers removed, were available for women with a mammogram recorded by BreastScreen SA from 1/10/2013 to 30/9/2016. They resided in 180 SA2 areas recorded in the ABS census in 2016. Of these, 172 SA2 areas were within South Australia where BreastScreen SA had recorded descriptive data on screening participation since the commencement of the screening program in 1991. Data collection was governed by nationally uniform data quality and consistency standards for reporting BreastScreen service delivery [3].

Data items included the following: Date of screening, date of birth, residential and/or postal address, and name of screening service attended. SA-NT Datalink, the principal data linkage service in SA, was used to geolocate individual client records. As client addresses had been manually entered into the BreastScreen database, data cleaning was undertaken to address any spelling errors in suburb names and street types, and flag imprecise “care of” addresses, post office box numbers, and interstate postcodes. The Quantum Geographic Information System (QGIS) was used to assign coordinates to individual client records [22], so that they could be mapped to the 2016 Australian Standard Geographical Classification (ASGC) SA2 areas of SA. Records with insufficient detail to be assigned coordinates by QGIS were provided with these coordinates through matching post-box addresses with a look-up table provided by Australia Post or through manual allocation using Google Maps.

**Fig. 1:**
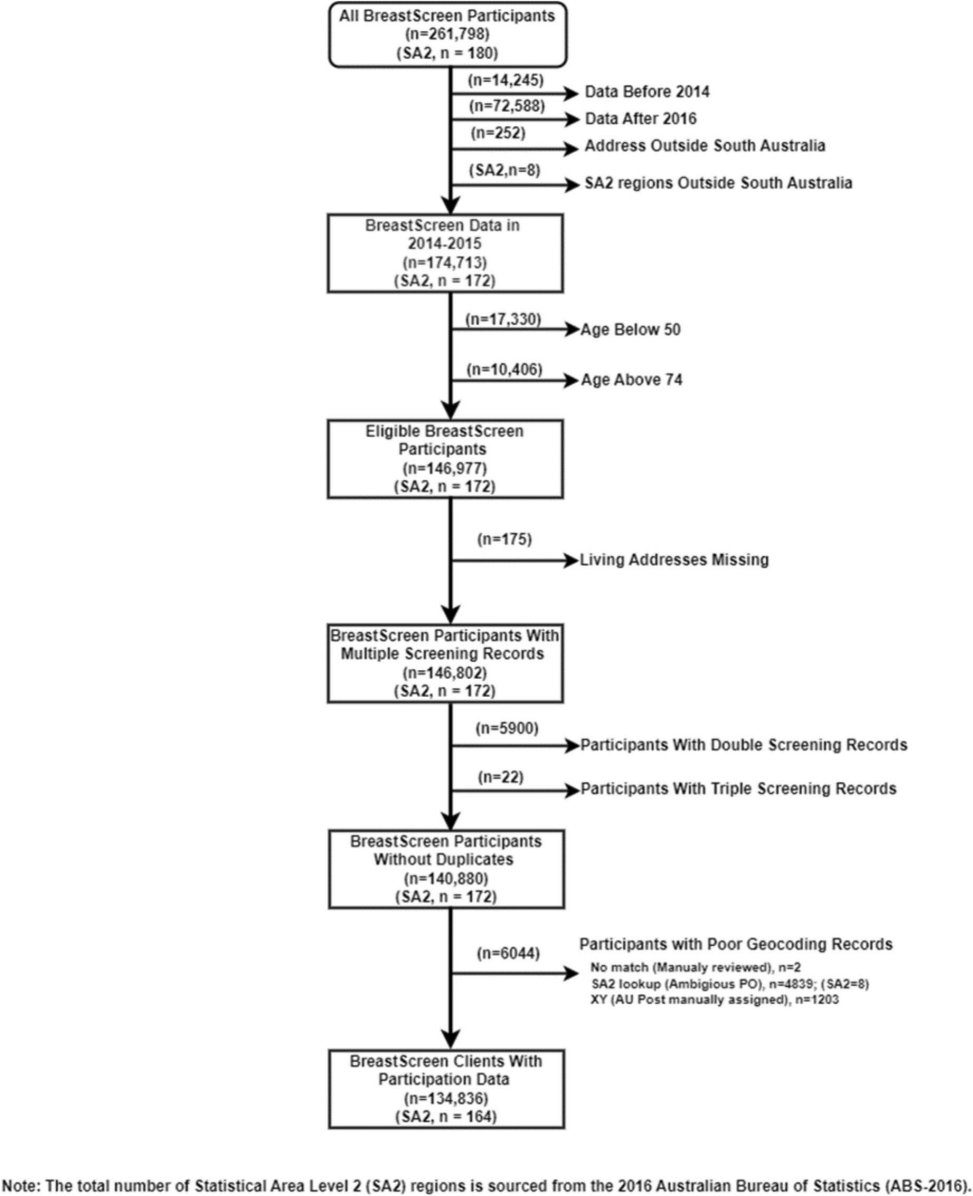
Population attending BreastScreen SA in 2014–2015. This flow diagram illustrates the population attending BreastScreen SA during the 2014–2015 period

The following inclusion criteria were applied for this study: 1. Screening between 1st Jan 2014 and 31st Dec 2015; 2. Having a residential address within a South Australian SA2 area when screened; 3. Being aged of 50–74 years at screen; 4. Having details of the latest screening episode where multiple screening episodes were recorded; and 5. Having a SA residential address with a valid geocode. The total number of final records/participants meeting these criteria was 134,836, covering 164 SA2 areas (Fig. 1).

### Study outcome

BreastScreen SA participation rates were calculated for the purposes of this study using total numbers of women screened at a BreastScreen SA service (the numerator) and corresponding numbers of eligible women aged 50–74 years estimated from the 2016 ABS census (the denominator). The participation rate, as calculated for this study, was the number of women screened (“x”) divided by the corresponding total number of women residents indicated by the ABS 2016 census (“y”), as “x/y.” This approach was used to obtain screening participation rates overall and by age for SA2 areas.

### Study factors (candidate indicators of screening participation)

SA2 area sociodemographic variables available as potential indicators of low screening participation included the following: Proportions of eligible women by country of birth, mothers’ educational attainment, employment status, whether unpaid care was provided to people with disability (UNCAREP), experience of mortgage/rental stress in low-income households, residential geographic remoteness, and the Index of Relative Socio-Economic Disadvantage (IRSD). Socio-demographic data at SA2 level were obtained from the ABS census through mapping undertaken by the Public Health Information Development Unit (PHIDU) at Torrens University [21].

Country of birth was expressed as the percentage of women who were Australian born [23]. Mother’s education attainment at SA2 level was classified according to whether year-10 schooling was completed by the age of 16 years [24]. Employment status was expressed as the percentage employed (full or part time) in the SA2 workforce [25]. UNCAREP was indicated by the percentage of people self-reported in the SA2 area as having provided unpaid care to people with disabilities/aged in the past two weeks [26]. Mortgage/rental stress in low-income households in SA2 areas was inferred from the percentage of low-income households paying > 30% of their income on housing costs [27]. The Socio-Economic Index for Areas (SEIFA) Index of Relative Socio-economic Disadvantage (IRSD) was expressed in quintiles derived from residential area postcodes [28]. Residential remoteness was classified according to whether the SA2 area was located predominantly in a major city area or inner regional, outer regional, remote or very remote area, as used by the Australian Standard Geographical Classification (ASGC) Remoteness Index [29].

### Statistical analysis

The unit of analysis in this study was the SA2 area. Screening participation rates were mapped using ESRI ArcGIS Pro version 3.1.2 for each of the 164 SA2 areas. Sociodemographic characteristics of SA2 areas were compared between those with participation rates above and below the state-level benchmark of 58%, using Chi-square tests for categorical variables and *t* tests or ANOVA for continuous variables, where appropriate. These comparisons are summarized in Table 1.

**Table 1:** Sociodemographic indicators at SA2 level for BreastScreen participation in 2014–2015 for women aged 50–74 years in South Australia

SA2-level indicators	SA2 areas with participation above state level *n* = 41	SA2 areas with participation below state level *n* = 123	Total (*n* = 164)	Participation rate (%)	*P* value
Mean age at screen (years) (SD)	61.2 (1.0)	61.2 (0.8)	61.2 (1.1)	50.6	0.859
% eligible women in the area	31.9 (0.05)	31.2 (0.06)	31.4 (0.06)	50.6	0.130
Country of birth tertile (% of Australia born)					0.020*
1st (38.7–70.7%)	11 (26.8)	44 (35.8)	55 (33.5)	52.3	
2nd (70.8–78.3%)	21 (51.2)	34 (27.6)	55 (33.5)	53.8	
3rd (78.8–91.5%)	9 (22.0)	45 (36.6)	54 (32.9)	45.4	
SEIFA^a^ quintile (%)					< 0.001*
1st (the least disadvantaged)	22 (55.0)	12 (9.8)	34 (20.9)	58.0	
2	8 (20.0)	23 (18.7)	31 (19.0)	52.5	
3	3 (7.5)	29 (23.6)	32 (19.6)	47.2	
4	4 (10.0)	32 (26.0)	36 (22.1)	48.2	
5th (the most disadvantaged)	3 (7.5)	27 (22.0)	30 (18.4)	46.2	
Remoteness					< 0.001*
Metropolitan	27 (65.9)	62 (50.4)	89 (54.3)	53.8	
Non-metropolitan	14 (34.1)	61 (49.6)	75 (45.7)	46.9	
% Mother education attainment < year 10					< 0.001*
1st (0–9.7%)	24 (58.5)	31 (25.2)	55 (33.5)	54.8	
2nd (10.1–16.4%)	12 (29.3)	43 (35.0)	55 (33.5)	49.8	
3rd (16.5–42.6%)	5 (12.2)	49 (39.8)	54 (32.9)	46.9	
Employment rate (%)					0.003*
1st (22.6–43.6%)	7 (17.1)	48 (39.0)	55 (33.5)	47.7	
2nd (43.7–48.0%)	12 (29.3)	43 (35.0)	55 (33.5)	50.7	
3rd (48.1–57.7%)	22 (53.7)	32 (26.0)	54 (32.9)	53.1	
% Unpaid disability care support					0.001*
1st (0–9.7%)	9 (22.0)	46 (37.4)	55 (33.5)	47.8	
2nd (9.8–10.5%)	15 (36.6)	40 (32.5)	55 (33.5)	52.9	
3rd (10.6–12.9%)	17 (41.5)	37 (30.1)	54 (32.9)	50.7	
% mortgage/rental stress in low-income households					0.001*
1st (5.3–21.5%)	18 (43.9)	37 (30.1)	55 (33.5)	47.6	
2nd (21.7–27.1%)	15 (36.6)	40 (32.5)	55 (33.5)	53.6	
3rd (27.2–57.1%)	8 (19.5)	46 (37.4)	54 (32.9)	50.3	

The average BreastScreen SA rate in 2014–2015 being 58% [24]; *P* value from *t* test for age at screen; chi-square test for other categorical variables. *Statistically Significant values

^a^SEIFA: Socio-Economic Index for Areas (SEIFA) missing for Lonsdale in ABS 2016; remoteness grouped into metropolitan as major cities, and non-metropolitan as inner regional, outer regional, remote, and very remote areas

Participation rates were compared and tested statistically using the *t* test, Chi-square tests, or instead ANOVA for variables with more than two sub-groups. Potential SA2 area indicators of low screening participation included percentages Australia born, percentages of mothers with low education attainment (< 10-year secondary school level), percentages employed, percentages of self-reported UNCAREP, and percentages of low-income households with mortgage/rental stress, with grouping into tertiles.

The BreastScreen SA participation rate was categorized into two groups according to whether or not the SA overall biennial participation reported by the AIHW of 58% (2014–2015) was achieved [1]. The odds ratio (95% CI) for not achieving this SA participation rate (i.e., having a low participation) was analyzed using logistic regression. In the multivariable analysis, sociodemographic indicators of screening odds were analyzed using backward stepwise elimination, with variable sequentially removed based on a threshold of *p* > 0.05 [30]. All variables that showed evidence of association in univariable analyses were initially included in the full model. Due to the small sub-group numbers by SEIFA IRSD quintile and remoteness categories, regroupings were done in the multivariable analysis as shown in the tabular output. Spatial autocorrelation of the breast screening participation rate across SA2 areas was assessed using Global Moran’s I, based on Queen contiguity spatial weights [31]. This test evaluates whether participation rates in geographically proximate areas are more similar than would be expected by chance. We followed the research reporting guidelines of the Strengthening the Reporting of Observational Studies in Epidemiology (STROBE) Statement for observational research [32].

All statistical analyses were conducted using STATA 17.0 (StataCorp. 2021. Stata Statistical Software: Release 17. College Station, Tx: StataCorp LLC).

## Results

### Characteristics of the SA2 areas

A total of 164 of 172 SA2 areas in SA were included in the study (95.3%), after applying the inclusion criteria (Fig. 1). Almost a third (31%) of women in the included areas were age-eligible for screening through the BreastScreen SA program, 74% were born in Australia, and about 21% resided in the least disadvantaged areas. Over half (54%) resided in a major city area. The overall employment rate was 45%. Mothers classified as having a low education level comprised 14%. The proportion providing unpaid care to people with disabilities/aged was 10%. The proportion having mortgage/rental stress in low-income households was 24%.

### BreastScreen SA participation rate

**Fig. 2:**
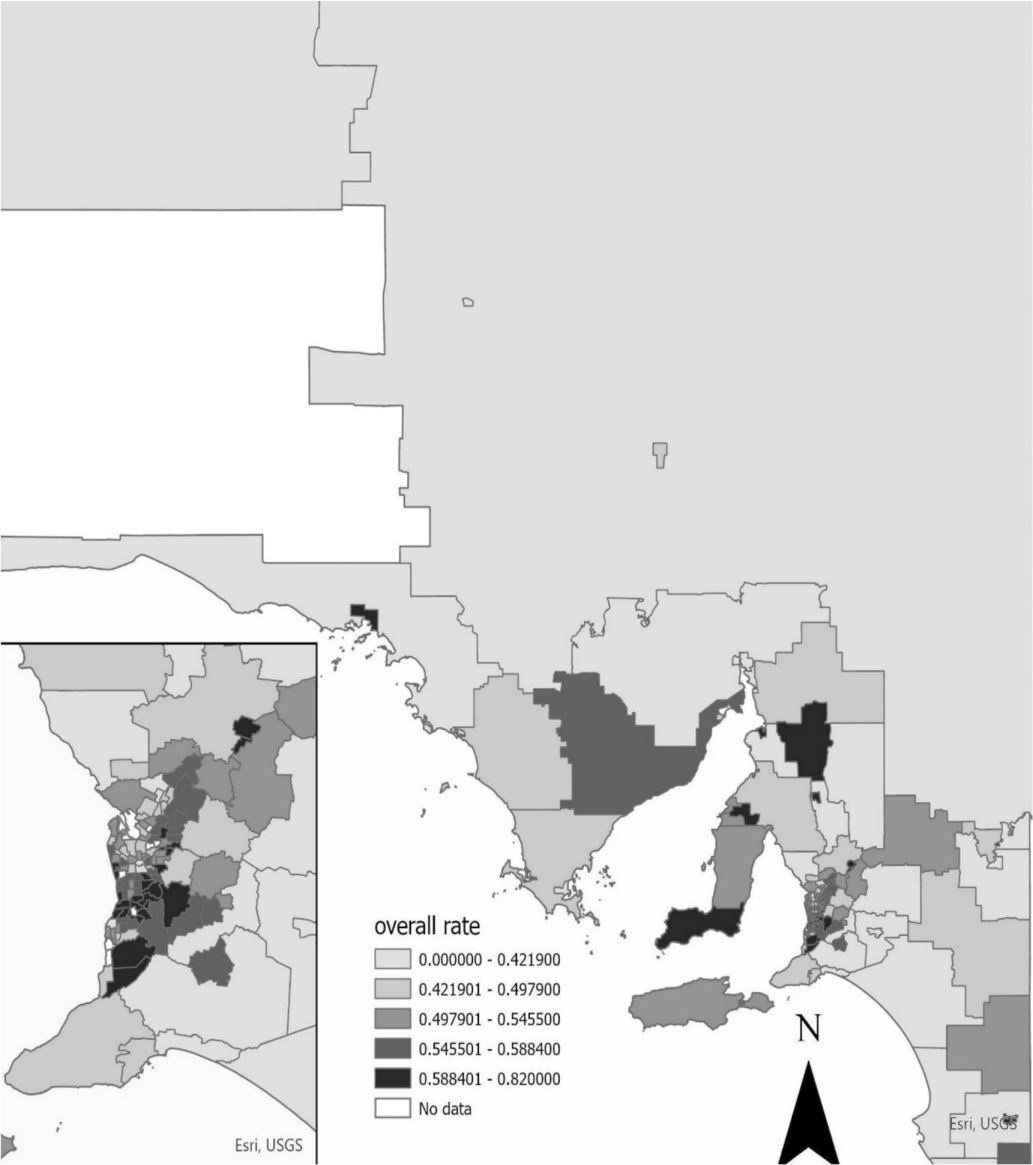
Participation rate of BreastScreen SA in 2014–2015. The white area on the map delineates a conservation park bordering Western Australia. This region is dedicated to natural preservation and devoid of residential properties, leading to an absence of data for measuring breast cancer screening participation. In addition, there are eight SA2 regions with no available data in the Breastscreen, namely Dry Creek, Western, Lonsdale, Torrens Island, Parafield, Flinders Ranges, Migratory-Offshore-Shipping, and Whyalla-North

The overall 2-year participation rate for the 164 SA2 areas included in the study was 50.6% (SD10.8; IQR 45.2–57.9) in 2014–2015 for women aged 50–74 years. Figure 2 shows participation rates across SA2 areas. Global Moran’s I was -0.03 (*p* = 0.269), indicating no statistically significant spatial autocorrelation in screening participation across SA2 areas. The map indicates participation to be higher in metropolitan areas than in regional and remote areas. Within metropolitan areas (Fig. 2 inset), the participation was higher in suburbs surrounding the Adelaide central business districts (CBD), especially in the south, south-east, and north-east. To further illustrate the spatial distribution of breast screening participation, a supplementary map was generated showing all SA2 areas classified by screening rates below and above the 58% threshold. Areas with low participation are highlighted in orange, while those above the threshold appear in light gray. This visualization highlights distinct geographic clusters of low participation across South Australia (Supplementary Fig. 1).

### Comparison of SA2 characteristics by screening rate

Sociodemographic characteristics of SA2 areas were compared based on whether breast cancer screening participation was above or below the South Australian benchmark of 58%, using Chi-square tests. Age-specific screening participation rates varied across the five-year age bands within the eligible population. Participation was lowest in the 55–59 age group (41.1%) and highest in the 60–64 age group (67.9%). Other age groups had moderate rates, with 49.1% for ages 50–54, 46.0% for 65–69, and 49.2% for 70–74 years. The mean age did not vary according to whether the SA2 participation was above or below the SA participation rate (Table 1). However, the following sociodemographic variables showed unadjusted differences in SA2 participation (*p* < 0.05): 1. Country of birth expressed as the percentage of Australian born: Areas with higher percentages of Australian born (≥ 78.8%) had a lower participation rate of 45.4% compared with areas with a lower percentage of Australian-born area (≤ 70.7%), which had a participation rate of 52.3%; 2. SEIFA quintile: The participation rate in the most disadvantaged areas was 46.2% compared to the least disadvantaged area of 58.0%; 3. Remoteness: The participation rate was 53.8% in metropolitan areas, which was higher than the 46.9% for non-metropolitan areas; 4. Mothers having low education attainment: In areas with a higher proportion of lower education attainment, the screen participation rate was lower (46.9% for the 3rd tertile and 49.8% for the 2nd tertile compared with 54.8% for the 1st tertile); 5. Employment: Areas with a higher employment rate had higher screening participation (53.1% in areas with the highest percentile employment 3rd tertile compared with 47.7% in the lowest percentage employment tertile); 6. Unpaid care provided to disabled/aged people (UNCAREP): The screen participation was higher in areas with a higher percentage of people providing unpaid care (50.7% for the 3rd tertile compared with 47.8% for the 1st tertile); 7. Mortgage/rental stress in low-income households: Areas with a higher percentage showing stress had higher screen participation (50.3% for the 3rd tertile compared with 47.6% for the 1st tertile) (Table 1).

In summary, using the BreastScreen SA total participation rate of 58% for 2014–2015, as reported in AIHW national breast screen report as the benchmark [23], a total of 41 SA2 areas achieved this participation (25.0%), while 123 (75.0%) did not. Areas not achieving this benchmark participation had a higher proportion of Australian-born residents (third tertile), more socio-economically disadvantaged residents, and more residing in non-metropolitan areas. In addition, areas not achieving the SA overall participation rate had higher proportions with low education attainment, more unemployment, and less evidence of mortgage/rental stress in a low-income household, while the proportion providing unpaid care to disabled/aged people was lower (Table 1).

### SA2-level sociodemographic indicators of BreastScreen SA participation

The univariable logistic regression showed the odds of not meeting the SA breast screening rate were lower in areas: With a proportion of 70.8–78.3% Australian-born population (OR: 0.38, 95% CI: 0.26–0.56) (Table 2). Also, compared with the reference category, the odds of lower screening participation were as follows: Highest in the most disadvantages area at OR: 16.06, 95% CI: 10.45–24.68; higher in the non-metropolitan areas at OR:1.84, 95% CI: 1.32–2.56; highest where mother’s education was lowest at OR: 7.59, 95% CI: 2.62–21.97; lowest where the employment rate was highest at OR: 0.19, 95% CI: 0.12–0.30; lowest where the percentage providing unpaid disability care support was highest at OR: 0.39, 95% CI 0.26–0.60; and highest where the percentage experiencing mortgage/rental stress was highest at OR: 3.04, 95% CI: 1.98–4.68 (Table 2).

**Table 2:** Odds ratios (95% CI) of SA2-level sociodemographic indicators for low BreastScreen participation in women aged 50–74 years in 2014–2015 in South Australia

SA2-level indicators	Unadjusted OR^†^ (95% CI)	Adjusted OR^†^ (95% CI)
% of eligible woman in the area	0.12 (0.01–1.89)	
Country of birth (% Australia born)		
1st (38.7–70.7%)	1.0	
2nd (70.8–78.3%)	0.38 (0.26–0.56)	0.52 (0.30–0.88)
3rd (78.8–91.5%)	1.17 (0.75–1.82)	0.27 (0.11–0.67)
SEIFA^a^ Index of Relative SES		
1st (least disadvantaged)	1.0	
2	5.23 (3.24–8.43)	6.08 (3.45–10.70)
3 4 5^ths^ (most disadvantaged)	16.06 (10.45–24.68)	17.00 (9.84–29.36)
Residential remoteness		
Metropolitan	1.0	
Non-metropolitan	1.84 (1.32–2.56)	4.94 (2.30–10.60)
% Mother education attainment < year 10		
1st (0–9.7%)	1.0	
2nd (10.1–16.4%)	2.77 (1.21–6.38)	
3rd (16.5–42.6%)	7.59 (2.62–21.97)	
Employment rate		
1st (22.6–43.6%)	1.0	
2nd (43.7–48.0%)	0.48 (0.30–0.77)	
3rd (48.1–57.7%)	0.19 (0.12–0.30)	
% Unpaid disability care support		
1st (0–9.7%)	1.0	1.0
2nd (9.8–10.5%)	0.49 (0.32–0.75)	0.41 (0.24–0.70)
3rd (10.6–12.9%)	0.39 (0.26–0.60)	0.81 (0.47–1.39)
% Mortgage/rental stress in low-income households		
1st (5.3–21.5%)	1.0	1.0
2nd (21.7–27.1%)	1.30 (0.91–1.89)	2.92 (1.68–5.07)
3rd (27.2–57.1%)	3.04 (1.98–4.68)	6.59 (3.34–13.00)

^**†**^OR from logistic regression analysis

^a^SEIFA: Socio-Economic Index for Areas, Index of Relative SES; remoteness grouped into metropolitan as major cities, and non-metropolitan as inner regional, outer regional, remote, and very remote areas

The socio-economic characteristics retained in the final multivariable analysis as indicators of low breast screening participation included SA2 areas with the following: Lower percentages of Australian-born residents; higher proportions of socio-economically disadvantaged individuals; more non-metropolitan residents; fewer individuals providing unpaid disability care support, particularly in the second tertile, which showed the strongest and statistically significant association with lower screening participation (OR: 0.41, 95%CI: 0.24–0.70), while the third tertile showed a weaker and non-significant association; and higher levels of mortgage/rental stress among low-income households. Mother’s education and employment status were excluded from the model when the other indicators were included. The indicators included in the multivariate model explained 28.2% of the variation in BreastScreen SA participation across these SA2 areas (Table 2).

## Discussion

BreastScreen SA participation was found to be 51% for the 164 SA2 areas included in this study, with variations indicating lower participation in the remote northern areas of SA. By comparison, metropolitan areas in the south, south-east, and north-east surrounds of the Adelaide CBD had higher participation. The participation rate for the 164 SA2 areas was lower than the total figure of 58% for SA overall provided by the AIHW for the same period [1]. This is attributed to inclusion criteria for the 164 selected SA2 areas, effects of differences in geocoding residential addresses at screening venues, and differences in denominator populations through use of the 2016 census.

The observed participation rate fell significantly below the national goal of 70% as previously observed [3]. This goal has never been achieved at an Australian jurisdiction level, despite strenuous efforts to do so. The challenges in achieving this goal are attributed in part to some women opting for mammography services in the private sector as an alternative screening option [33]. The notably higher participation in the 60–64 age group compared to other age bands may reflect greater health engagement or more targeted outreach during this life stage. Conversely, the low participation among women aged 55–59 could indicate transitional life factors (e.g., workforce demands, caregiving roles) that impact screening uptake. These intra-cohort differences underscore the value of targeted communication and service delivery strategies tailored to age-specific barriers, even within an ostensibly uniform eligibility band.

Variations in screening by SA2 area point to areas of low screening that may warrant further consideration in ongoing screening promotion. Multivariable analyses indicate that these areas are characterized by higher proportions of women born outside of Australia, higher proportions of socio-economically disadvantage women, higher proportions living outside of metropolitan areas, and more experiencing mortgage/rental stress in low-income households. By comparison, SA2 areas with higher percentages providing unpaid disability/aged care support did not show a linear association with screening participation, with the middle range tertile being less likely to have a low breast screening percentage than the least affected SA2 tertile.

The findings of this study align with Andersen’s Healthcare Utilization Model [20]. For example, predisposing factors such as country of birth and educational attainment, and enabling factors like socio-economic status and remoteness, were significantly associated with lower screening participation. This reinforces the role of both individual and contextual characteristics in shaping health service utilization, particularly in the case of organized screening programs. Applying this framework also highlights how local-level structural and social determinants interact to produce persistent spatial disparities.

Multivariable analyses indicated SA2 participation profiles by socio-economic and residential remoteness to be consistent with the following: Person-level breast screen participation data recorded nationally [1], area-level analyses in Victoria [18], and with person-level data for patients with breast cancer in NSW, SA, and Queensland [4, 5, 7, 9]. The multivariable results of lower participation in SA2 areas with higher proportions of non-Australian-born residents may reflect cultural differences that need further attention in breast screening promotion. A prior study indicated that some women of culturally diverse background had competing cultural beliefs and attitudes and sometimes had limited English proficiency potentially presenting communication barriers [34]. Additionally, another study of culturally diverse women in NSW reported the absence of symptoms, fatalistic beliefs, and embarrassment associated with the screening procedure to be primary reasons for reluctance to be screened [35]. Further research indicated that the lack of a general practitioner (GP) endorsement, transport issues, and anticipated pain associated with the procedure could present additional barriers to screening participation [35].

Multivariable analysis results were plausible in indicating that SA2 areas with low screening participation had higher mortgage/rental stress in low-income households [27]. Such households potentially have poorer affordability of life’s necessities such as food, healthcare, and education. Education and wealth have been demonstrated to have strong negative associations with poor health in large, multi-country cohorts, including in Australia [36]. Over a 20-year period, similar results from the Organization for Economic Cooperation and Development (OECD) countries confirm education levels to be impactful in improving self-awareness of personal health and increasing access to health services [37]. Furthermore, low-income has been reported as one of the most important factors associated with lower breast cancer screening [38], with a USA study of women’s views indicating that barriers were imposed by the lack of paid time-off, limited transportation, fear, and lack of support to accommodate scheduling requirements [33]. In addition, women commonly reported psychological obstacles such as fear of high cost, fear of mammogram-associated pain, and fear of receiving bad news [33]. Our results and literature review support the need to continue ongoing educational and outreach initiatives in low-income communities [39].

An unexpected observation in the present study was the suggestion of a higher provision of unpaid care for disabled and aged people occurring in SA2 areas with a higher likelihood of screening participation, although the gradient was not consistent. This observation aligns with inconsistent findings in the existing literature [40, 41]. For example, a study in Ireland, using linked breast screening and census data, indicated that women serving as carers for < 20 h/week were more likely to attend breast screening [40], whereas another Australian study indicated that women aged 50 years and over with caregiving responsibilities had lower participation in health promoting activities compared to women without these caregiving responsibilities [41]. Despite inconsistencies in the evidence, it is possible that some caregivers in the present study were more protective of their health due to self-selection factors associated with being caregivers. Further investigation is warranted to explore potential confounding influence of individual factors such as socio-economic status, time availability, workload, and health knowledge.

The present results indicate the potential value of SA2 characteristics in SA to affect likelihood of breast screening participation. In particular, standout results from the multivariable analysis were the lower screening participation in non-metropolitan areas, and areas characterized by more non-Australian born, and more socio-economic disadvantage, less evidence of provision of unpaid care support for disabled people, and more evidence of mortgage/rental stress in low-income households. These screening participation indicators accounted for 28% of area disparities in participation.

A strength of the study was use of screening participation data provided by BreastScreen SA, which are thought to be more accurate and reliable than self-reported data. Study limitations in the area-based analysis included exclusion of personal factors were not addressed directly. Breast screening participation is an individual behavior that may be influenced by person-level factors such as health literacy and psychosocial characteristics. Geospatial environmental factors may also be important, however, including distance to health services [16], general practitioner availability [36], and transport options [39]. The proportion of residents with Culturally and Linguistically Diverse (CALD) backgrounds has long been identified as a negative correlate of screening participation [2]. This is a culturally heterogeneous group, however, which requires more detailed study at sub-group level of potential CALD determinants, such as those available through use of the newly available Person Level Integrated Data Asset (PLIDA) platform that include proficiency in spoken English, ancestry, and other cultural characteristics of individuals and parents [16].

Although inclusion of additional contextual variables (e.g., healthcare provider availability or proportion of eligible women) could further strengthen the analysis, such data were not available at the SA2 level from our sources. Additionally, the use of multilevel modeling was not feasible given the aggregate nature of our data and the absence of individual-level records. SA2 units remain a valid and policy-relevant spatial scale for examining screening disparities, as they reflect meaningful local variation defined by the Australian Bureau of Statistics.

A number of initiatives undertaken in SA to address potential geospatial barriers have included the Government-funded Patient Assistance Transport Scheme (PATS) operating in six regional local health networks by the Rural Support Service of SA Health [42]. Another example is the deployment by BreastScreen SA of mobile screening units for women living in outer-metropolitan, rural, and remote regions at 36 locations across South Australia every 2 years [43]. It is important to further evaluate the effectiveness of these programs in countering distance barriers.

Another study limitation was the potential for a significant underestimation of screening participation in areas where women may access private medical settings rather than participating in the national BreastScreen Program [10]. Data on private sector screening uptake are not routinely captured at the SA2 level, limiting our ability to quantify this effect. If private sector use varies geographically—e.g., with higher use in more affluent or urban areas—this could lead to differential underestimation of screening rates and potentially bias area-level comparisons. Some underestimations also would result from use of the 2016 census as the denominator population since this would have exceeded the population size in 2014–2015.

While our data were collected prior to the COVID-19 pandemic, the core socio-economic and geographic factors influencing screening participation—such as country of birth, socio-economic disadvantage, remoteness, and access to healthcare—are unlikely to have shifted dramatically due to the pandemic. Moreover, within South Australia, BreastScreen services have remained stable, with minimal changes to clinic locations or operating h, and no substantive modifications to the invitation process. This operational consistency supports the ongoing relevance of our findings in understanding structural and contextual influences on screening participation. However, we acknowledge that changes in healthcare utilization patterns, particularly related to shifts in health-seeking behavior, public health messaging, and service delivery, may affect current screening dynamics. The study’s insights based on pre-pandemic data remain valuable for understanding the broader, persistent drivers of screening disparities. Nonetheless, the findings should be interpreted with caution when applying them to the present, and future research with more recent data is needed to evaluate whether these historical trends still hold in the post-pandemic context.

Finally, data availability for the study at SA2 areas was insufficient to include all 172 SA2 areas, with potential for the 164 (95%) that were included not to be entirely representative.

In summary, despite study limitations, the SA2 characteristics found to be associated with screening participation were plausible. It is likely that screening participation is affected by sociodemographic characteristics of the women themselves, together with characteristics of the areas in which they live. Broader analyses drawing conjointly on a broader range of person-level and small-area-level screening indicators may best explain variations in screening participation for informing service plans. Also, areas with most unexplained variations could be identified more effectively through these broader analyses for further research. An ideal option may be to re-run geospatial analyses periodically to coincide with subsequent censuses. In that event, the present data could serve as an earlier benchmark.

In conclusion, Breast screening participation has plausible associations with sociodemographic characteristics of the SA2 areas in which SA women lived. Further model development should explore the best model fit when combining characteristics from a person level and residential area level to indicate extent of unexplained variation. CALD characteristics could be further explored at sub-group level using the newly available ABS PLIDA platform. Also, progressively more contemporary census data should be used in prospective monitoring.

## Supplementary Information

Below is the link to the electronic supplementary material.

Supplementary file1 (DOCX 120 KB)

## Data Availability

The breast screening data are confidential and subject to privacy laws. Access to these data may be obtained on request from BreastScreen SA but it is not publicly disseminated.

## Abbreviations

ABS: Australian Bureau of Statistics
ASGC: Australian Standard Geographical Classification
CBD: Central Business Districts
CALD: Culturally and Linguistically Diverse
COVID-19: COronaVIrus Disease of 2019
GP: General Practitioner
IRSD: Index of Relative Socio-Economic Disadvantage
IQR: Interquartile Range
NAS: National Accreditation Standards
NBCF: National Breast Cancer Foundation
NSW: New South Wales
OECD: The Organization for Economic Cooperation and Development
PATS: Patient Assistance Transport Scheme
PHIDU: Public Health Information Development Unit
PLIDA: Person Level Integrated Data Asset
QGIS: Quantum Geographic Information System
SA: South Australia
SA2: Statistical Areas Level 2
SD: Standard Deviation
SEIFA: The Socio-Economic Indexes for Areas
UNCAREP: Unpaid care provided to disability/aged people
