# Supplementary material for: Determinants of breast screening participation using small-area data in South Australia: gaining past and future insights from geospatial evidence
Source: Cancer Causes Control. 2025 May 14;36(10):1107–18. doi: 10.1007/s10552-025-02009-z (PMC12578701; doi:10.1007/s10552-025-02009-z)

**Supplementary Figure 1:** Small-area variation in breast screening participation across Statistical Area Level 2 (SA2) regions in South Australia. Areas with low participation are highlighted in orange. This visualization highlights distinct geographic clusters of low participation across South Australia


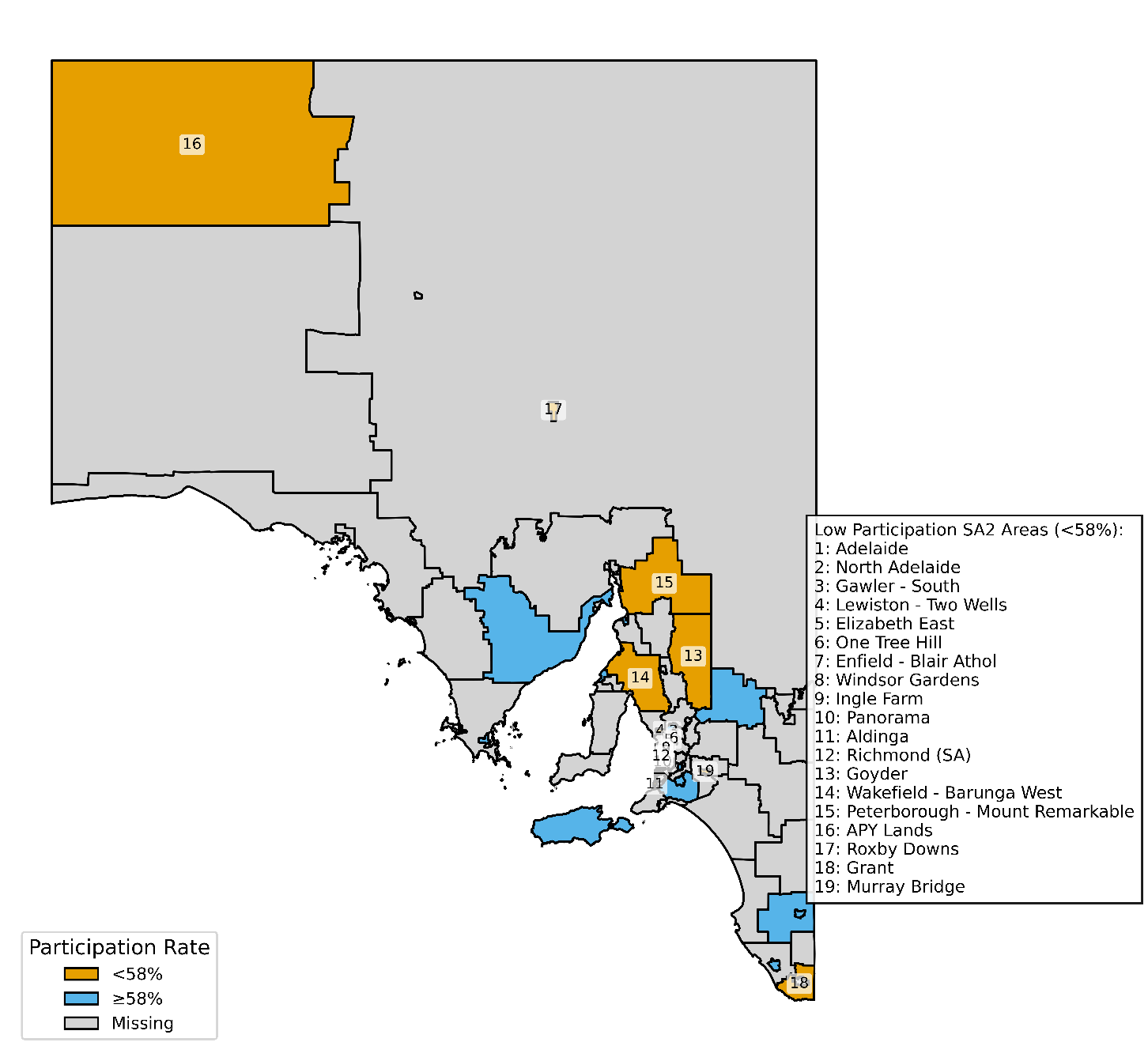

Supplement: Supplementary file 1 — Supplementary file1 (DOCX 120 KB) [file 10552_2025_2009_MOESM1_ESM.docx]
